# Comparative performance evaluation of hepatitis C virus genotyping based on the 5' untranslated region versus partial sequencing of the NS5B region of brazilian patients with chronic hepatitis C

**DOI:** 10.1186/1743-422X-8-459

**Published:** 2011-10-03

**Authors:** Sueli M Nakatani, Carlos A Santos, Irina N Riediger, Marco A Krieger, Cesar AB Duarte, Maria do Carmo Debur, Flair J Carrilho, Suzane K Ono

**Affiliations:** 1 Laboratório Central do Estado do Paraná, LACEN-PR, São José dos Pinhais, Paraná, Brazil; 2 Centro de Genomas, São Paulo, São Paulo, Brazil; 3 Instituto Carlos Chagas (ICC) FioCruz, Curitiba, Paraná, Brazil; 4 Department of Gastroenterology, São Paulo University School of Medicine, São Paulo, Brazil

**Keywords:** HCV, genotyping, NS5B region, 5'UTR

## Abstract

**Background:**

Genotyping of hepatitis C virus (HCV) has become an essential tool for prognosis and prediction of treatment duration. The aim of this study was to compare two HCV genotyping methods: reverse hybridization line probe assay (LiPA v.1) and partial sequencing of the NS5B region.

**Methods:**

Plasma of 171 patients with chronic hepatitis C were screened using both a commercial method (LiPA HCV Versant, Siemens, Tarrytown, NY, USA) and different primers targeting the NS5B region for PCR amplification and sequencing analysis.

**Results:**

Comparison of the HCV genotyping methods showed no difference in the classification at the genotype level. However, a total of 82/171 samples (47.9%) including misclassification, non-subtypable, discrepant and inconclusive results were not classified by LiPA at the subtype level but could be discriminated by NS5B sequencing. Of these samples, 34 samples of genotype 1a and 6 samples of genotype 1b were classified at the subtype level using sequencing of NS5B.

**Conclusions:**

Sequence analysis of NS5B for genotyping HCV provides precise genotype and subtype identification and an accurate epidemiological representation of circulating viral strains.

## Background

The Hepatitis C virus (HCV) genome sequence is highly variable. Six major types and approximately 80 subtypes have been recognized since it was first identified [1]. The nucleotide level differs by 31% to 33% among genotypes and by 20% to 25% among subtypes [2]. Genetic variation throughout the genome is not uniform. The region encoding envelope glycoproteins was the most variable when compared to the highly conserved 5' untranslated region (5'UTR) [3]. Most of the commercially available genotyping methods are based on the detection of the conserved bases within the 5'UTR region. However, the ability of the 5'UTR nucleotide sequence to discriminate virus isolates at the subtype level is controversial, and alternative regions have been proposed for genotyping [4]. The widely accepted reference method for HCV genotyping is the NS5B region sequencing [5]. Therefore, the aim of the present study was to compare a genotyping method based on partial sequencing of the NS5B region to a commercial method based on the 5' UTR region (LiPA) using plasma samples obtained from Brazilian patients.

## Materials and methods

### Plasma samples

A total of 171 plasma samples representing HCV genotypes 1, 2, 3, 4, and 5 were used in this study. All samples had been previously genotyped by line probe assay (LiPA) v.1 using the Versant™ HCV Genotype Assay (Siemens, Tarrytown, NY, USA) after amplification of 244 bp of the 5'UTR fragment, which had been generated using the Amplicor^® ^Hepatitis C Virus (HCV) Test, version 2.0 (Roche, Branchburg, NJ, USA) according to the manufacturer's instructions.

This study protocol was approved by the Ethics Committee of the University of São Paulo (CAAE - 2546.0.015.000-05).

### RNA extraction

RNA extraction was performed using the NucliSENS Magnetic Extraction Reagents (bioMérieux, Boxtel, the Netherlands). A total of 200 ml of plasma was added to the lysis buffer and incubated for 10 minutes at room temperature. Magnetic silica particles were used for nucleic acid binding for 10 minutes at room temperature. Silica particles were washed with different buffers, and the NucliSENS miniMAG apparatus was used to collect and wash the particles. The nucleic acids were released from the silica particles using 60 ml of elution buffer and by heating the samples to 60°C for five minutes.

### RT-PCR

The synthesis of cDNA was performed essentially as previously described [6]. For reverse transcription, 40 ml of RNA was added to the reaction mixture [3 ml of random primers (7.5 ng/ml) and 36 ml of DEPC-H_2_O] and incubated at 70°C for 10 minutes. Then, 24 ml of Reverse Transcription mix [5X buffer, 0.1 M DTT, 10 mM dNTPs, 30 U/ml RNase Out, and 200 U/ml M-MLV RT (Invitrogen, Carlsbad, CA, USA)] were added. Reverse transcription was performed using the GeneAmp PCR Systems 9700 (Applied Biosystems, Foster, CA, USA) using the following conditions: 25°C for 15 min, 37°C for 62 min, 95°C for 15 min, and a final hold at 10°C.

### Amplification of the HCV cDNA

All primers described in this study were designed based on the NS5B region consensus sequences, which were obtained upon alignment of the data provided by the Los Alamos National Laboratory http://hcv.lanl.gov/content/sequence/HCV/ToolsOutline.html.

Table 1 includes details for the primers (Invitrogen) that were used in the PCR amplification of the NS5B region. Each PCR reaction contained 10X buffer [200 mM Tris-HCl (pH 8.4), 500 mM KCl], 1.5 mM MgCl_2_, 10 mM dNTPs, 1 μM of each primer, 2.5 UI of Platinum Taq DNA Polymerase High Fidelity (Invitrogen) and 5 μl of the cDNA to be tested in a final volume of 50 μl that was obtained using DEPC-treated H_2_O. PCR was performed in the GeneAmp PCR System 9700 (Applied Biosystems) using the following amplification conditions: 5 min at 94°C; 35 cycles at 95°C for 30 s, 60.5°C for 1 min for primer NS5B2F/NS5B2R, 56°C for 45 s for primer F56_1-3/R56_1-3, or 58°C for 45 s for primer GEN2FSN/GEN2RSN, and 72°C for 1 min; 72°C for 10 min; and a final hold at 10°C.

**Table 1:** PCR primers used in NS5B-based in-house methods

Primer name	Genotype	Sequence	Product	Position Region NS5B*
NS5B2F	All genotypes	5'-TTCACGGAGGCTATGACYAGG-3'	688 bp	1017-1038
				
NS5B-2R		5'-CGGGCATGMGACASGCTGTGA-3'		1704-1683
				
F56_1-3	1 and 3	5'-CACACTCCAGTYAAYTCCTGG-3'	241 bp	1204-1224
				
R56_1-3		5'-CWMCTGGAGAGTAACTGTGGAG-3'		1444-1423
				
GEN2FSN	2	5'-GACACTCCCCTGTCAATTCWTGG-3'	120 bp	1202-1224
				
GEN2RSN		5'-TGGTYCAGAGTGTCYTGGGC-3'		1321-1313

*1a_.H77.NC_004102 - reference sequence that was used to calculate the positions of the NS5B region.

### Nucleotide sequencing of NS5B region

PCR products were purified using the PureLink™ PCR Purification Kit (Invitrogen), quantified using the Kodak Digital Science Analysis System 120 (Rochester, NY, USA) and diluted to 20 ng/μl. Sequencing reactions were performed in both directions using the Big Dye terminator version 3.2 (Applied Biosystems), and the products were detected using the ABI 3130 Genetic Analyzer (Applied Biosystems).

### Sequence analysis

Nucleotide sequences from HCV strains were edited using Codon Code (Codon Code Corporation, Dedham, MA, USA) and imported to BioEdit [7], which was used to align the sequences to a reference panel of reported sequences provided by the Los Alamos National Laboratoryhttp://hcv.lanl.gov/content/sequence/HCV/ToolsOutline.html.

A total of 171 HCV NS5B sequences of different genotypes that were identified in this study were submitted to GenBank and can be retrieved under the accession numbers EF136859 to EF136882 and FJ159697 to FJ159845.

### Phylogenetic analysis

An internal fragment of 216 nucleotides within the PCR products that were generated for sequencing (positions 1223-1438 in the NS5B region according to the reference sequence H77, GenBank accession number NC004102) was used for phylogenetic analysis with 138 of the 171 (80.7%) samples. Nucleotide distances were computed with MEGA version 4.0 [8] using the ρ-distance algorithm. Phylogenetic trees were inferred using the neighbor joining method. Robustness of the tree branches was tested using bootstrap analysis (1000 replicates).

## Results

### HCV genome amplification

The primer pair NS5B2F/NS5B2R was efficient in amplifying most of genotypes 1 to 5. However, these primers failed to amplify six samples of genotype 2. To amplify these samples, we designed a specific primer pair (GEN2FSN/GEN2RSN). Among these amplified samples, five were classified as genotype 2 c and one as 2b.

### Performance comparison of HCV genotyping using 5'UTR (LiPA) and NS5B sequence analysis

Comparison of HCV genotyping was based on NS5B sequence analysis and LiPA (5'UTR) and showed no difference in the classification of all 171 samples (Table 2). However, discrepancies at the subtype level were found for 47.9% (82/171) of the subtyping results between LiPA and NS5B sequencing (Table 3). LiPA did not assign subtypes for 40 samples of genotype 1, which were classified as genotype 1a (34 samples) and genotype 1b (6 samples) by partial NS5B sequencing. Four samples that were classified by LiPA as genotype 1b were grouped as genotype 1a by NS5B sequencing. Among 26 genotype 2 samples, LiPA classified five samples as genotype 2b. Forty-two genotype 3 samples were correctly typed as 3a by LiPA, which was confirmed by NS5B sequencing.

**Table 2:** Comparison of HCV genotyping results from LiPA (5'UTR) and partial NS5B sequencing

LiPA type	Number of specimens with NS5B sequencing								Total
		
	1a	1b	2a	2b	2c	3a	4a	5a	
**1**	34	6							40
**1a**	10	3							13
**1b**	4	32							36
**1a/1b**	7	4							11
**2**			1	11	7				19
**2b**				5					5
**2a/2c**					2				2
**3a**						42			42
**3a/3b**						1			1
**4**							1		1
**5**								1	1

**Total**	**55**	**45**	**1**	**16**	**9**	**43**	**1**	**1**	**171**

**Table 3:** Clinical samples with discrepant subtyping results between LiPA(5'UTR) and partial NS5B sequencing

**No. of samples**	**LiPA (5'UTR)**			**NS5B sequencing**
		
	**Non-subtyped**	**Discrepant results**	**Inconclusive**	
		
34	1			1a
6	1			1b
4		1b		1a
3		1a		1b
7			1a/1b	1a
4			1a/1b	1b
1			3a/3b	3a
11	2			2b
7	2			2c
1	2			2a
2			2a/2c	2c
1	4			4a
1	5			5a

### Inconclusive genotypes

LiPA classified 11 samples as genotype 1a/1b, which were further subtyped as genotypes 1a (7/11) and 1b (4/11) by NS5B sequencing. Two samples yielded LiPA patterns that were categorized as genotype 2a/2c. However, NS5B sequencing subtyped these samples as genotype 2 c (Table 3).

### Phylogenetic analysis

Discrepancies were not found when the results of partial NS5B sequencing and phylogenetic analysis of 138 samples were compared.

## Discussion

Hepatitis C virus genotyping assays are usually based on the sequence analysis of an amplified segment of the genome, which is commonly the 5' untranslated region. Currently, 5'UTR based assays are reasonably accurate with more than 95% concordance with genotypes that have been identified by nucleotide sequencing of the NS5B region or other coding regions of the HCV genome [9]. In our study, LiPA and NS5B sequencing showed 100% agreement at the type level. These results are similar to those reported by another study using the 5'UTR with a type agreement of 99.5% [10]. Another study with 357 samples from French blood donors has demonstrated 100% agreement between 5'UTR and NS5B sequence analysis regarding type classification [11]. However, depending on the geographical region, genotype identification based on 5'UTR may be unreliable because some genotype 6 variants that have been found in Southeast Asia have identical 5'UTR sequences to those of genotypes 1a or 1b [12]. In Brazil, the most prevalent HCV genotypes are 1, 2 and 3, whereas genotypes 4 and 5 are rarely identified and genotype 6 has not been previously described [13].

When we compared the subtyping results of LiPA and partial NS5B sequencing, we found 47.9% (82/171) of misclassification among samples, including non-subtypable discrepant and inconclusive results. LiPA could not discriminate 39.6% (40/101) of the genotype 1 samples at the subtype level. LiPA misclassified three samples of genotype 1b as genotype 1a and four samples of genotype 1a as genotype 1b. LiPA did not accurately discriminate between genotypes 1a and 1b, which may be attributed to the lack of discriminating power of LiPA probes 5 and 6. The difference between these probes covers a single nucleotide change in the HCV genome, which is an A to G transition at position -99 within the 5'UTR. There is evidence that this transition represents a sequence polymorphism that cannot be used to differentiate between subtypes 1a and 1b [14]. Accordingly, we found that LiPA yielded inconclusive results for 11 genotype 1 samples, which were classified as genotype 1a/1b.

Concerning genotype 2, 26 samples were investigated in our study. The small sample size is due to the low prevalence of this genotype in Southern Brazil (less than 4%). In our study, only five of the 26 genotype 2 samples were simultaneously subtyped as 2b by LiPA and NS5B sequence analysis. The 21 remaining genotype 2 samples were not accurately subtyped by LiPA. After performing the sequence analysis of the NS5B region, 11 of these 21 samples were classified as genotype 2b, nine samples as genotype 2 c and one sample as genotype 2a. This poor resolution was expected because LiPA targets 5'UTR, which may result in incorrect identification of subtypes 2a and 2 c due to the lack of nucleotide polymorphisms [15]. Similarly, a previous study has reported that 61% of the subtypes of genotype 2 were misclassified by 5'UTR-based genotyping in France [11]. Another study has compared genotyping using real-time PCR targeting the NS5B region and LiPA v.1.0 and has shown that LiPA did not subtype 68 of the 295 samples (23%) [16].

The low reported prevalence of mixed HCV genotype infection suggests that this type of infection is a rare event and is influenced by the population that is studied and the genotyping method that is employed [17]. A study with 600 patients has shown that 2.2% of patients displayed evidence of mixed infections. These sera have been screened using sequencing and serological assays in parallel [18]. Accordingly, only two laboratories have correctly identified one of the three samples containing mixed HCV genotypes in a national evaluation study that was conducted in France [19]. Cases of mixed HCV genotypes have not been confirmed in our study. Although all of the samples were characterized as infected based on a single genotype, direct sequencing is not the gold standard method to detect mixed infection. The best approach to reliably detect mixed-genotype infections is PCR amplification followed by cloning of the PCR products or the use of next-generation sequencing methods. Unfortunately, this approach is unfeasible in a routine clinical setting [20]. The results that are reported herein are similar to those found in another study that has been conducted in Brazil using sequencing to genotype 1,688 samples from chronic HCV patients, who did not show evidence of mixed infection [21].

In our study, the 5'UTR-based genotyping method (LiPA) showed higher efficiency than the NS5B region to generate PCR amplification products. Primers targeting the 5'UTR, which is a highly conserved region, produce fewer false negative results in PCR reactions for HCV detection. The major factor to be considered when evaluating the generation of false negative PCR results is the existence of different strains or subtypes of HCV [22]. Because the polymorphic positions are not homogenously distributed within the NS5B region, difficulty to generate amplicons may be due to inadequate primer design, choice of highly polymorphic annealing sequences or low viral load [19].

Currently, there is a new available commercial version of LiPA (v.2), which contains probes targeting both the 5'UTR and the core regions of the HCV genome, which improves the accuracy of subtyping, particularly regarding the discrimination between subtypes 1a and 1b. By the time this study was conducted, this version was not available for use in Brazil.

In the future, a protective vaccine and the availability of HCV-specific antiviral drugs that are based on proteases or polymerase inhibitors will probably require genotyping at the subtype level. This genotyping approach will improve our understanding of the genetic diversity of HCV. Genotyping methods that are based on the 5'UTR are important tools for routine clinical purposes because they are sufficiently precise at the genotype level. Our results show that HCV genotyping using partial NS5B sequence analysis is an efficient method that allows accurate discrimination of subtypes and might be an effective tool to study the molecular epidemiology of HCV.

## Competing interests

The authors declare that they have no competing interests.

## Authors' contributions

SMN, CAS, and SKON conceived and designed the experiments; SMN and INR performed the experiments; SMN, CAS, and CABD analyzed the data; MAK and FJC contributed reagents/analysis tools; and SMN and INR wrote the paper. All authors read and approved the final manuscript.
